# A Successfully Treated COVID-19 Vaccine Induced Immune Thrombocytopenic Purpura

**DOI:** 10.7759/cureus.49878

**Published:** 2023-12-03

**Authors:** Miguel A Rodriguez Guerra, Siddharth Chinta, Ana P Urena Neme, Sorab Gupta, Gabriella Roa Gomez

**Affiliations:** 1 Medicine, Montefiore Medical Center, Albert Einstein College of Medicine, Bronx, USA; 2 Medicine, BronxCare Health System, Bronx, USA; 3 Cardiology, Medicina Cardiovascular Asociada, Santo Domingo, DOM; 4 Department of Hematology and Oncology, Einstein Healthcare Network, Philadelphia, USA; 5 Pulmonary and Critical Care Medicine, Albert Einstein College of Medicine, Bronx, USA; 6 Pulmonary and Critical Care Medicine, Montefiore Medical Center, Wakefield Campus, Bronx, USA

**Keywords:** intravenous immunoglobulin (ivig), vaccine induced immune thrombocytopenia, mrna covid-19 vaccine, bnt162b2 vaccine, covid 19

## Abstract

Immune Thrombocytopenic Purpura (ITP) is a life-threatening condition where an accurate initial assessment is essential to be able to offer the proper therapy in a timely matter to improve the outcome of the patient. Here, we present a case of ITP secondary to the coronavirus disease 2019 (COVID-19) vaccine (BioNTech, Pfizer vaccine). A 61-year-old obese African American female presented to the emergency room (ER) from a clinic with a platelet count of 11k/ul 21 days after she received the second dose of the BioNTech, Pfizer vaccine. The patient was immediately started on intravenous immunoglobulin (IVIG) 1g/kg twice daily (bid) and dexamethasone 20 mg IV every 12 hrs (q12h). The next day, the platelet count increased to 63 k/ul, and after the second dose of IVIG, the platelet count improved to 122 k/ul and trended up. The early detection of ITP induced by the mRNA COVID-19 vaccine is determinant to guide the early and proper therapy with immunoglobulins and steroids to improve the outcome of our patients.

## Introduction

Immune thrombocytopenic purpura (ITP) is an acquired condition characterized by immune-mediated destruction of platelets and impairment of platelet production. There can be several causes of ITP, including human immunodeficiency virus (HIV) and hepatitis infection [1]. Recently, it has been postulated that ITP can be precipitated by immunological reactions, including coronavirus disease 2019 (COVID-19) vaccination [1]. ITP is a diagnosis of exclusion, and its diagnosis is essential to be able to offer proper therapy in a timely manner [2]. 

As of June 10, 2021, the Centers for Disease Control reports that two hundred million people have been vaccinated in the United States with the COVID-19 vaccine [3]. These vaccinations confer various side effects and over 6,994 cases have been reported due to COVID-19 vaccines. Out of the total, 91% of the cases were not life-threatening and nonspecific; the most common adverse reactions are reported within the first 30 minutes after administering the vaccine [4]. Here, we present a rare case of ITP induced by BioNTech, Pfizer COVID-19 vaccine.

## Case presentation

We present a case of a 61-years-old African American female with a history of hypertension, mixed connective tissue disease, fibromyalgia, gastroesophageal reflux disease (GERD), prediabetes, cervical stenosis, asthma, iron deficiency anemia who was referred to our emergency room for a platelet count of 11 k/ul and anemia (hemoglobin (Hb): 6.7 g/dL, hematocrit (Hct) 20.9). The patient had been admitted to another institution about one week prior to admission for hematuria and urinary tract infection (UTI). At that time, she was found to have a platelet count of 8 k/ul. Further history revealed that she had received the second dose of BioNTech, Pfizer vaccine about 21 days prior to presentation to ER and 14 days prior to thrombocytopenia seen in other hospitals. She had bloodwork four days prior to the vaccine and was found to have a platelet count of 250 k/ul and had another complete blood count four days after the vaccine and was found to have a platelet count of 261 k/ul (Table 1). Physical examination was unrevealing for petechiae, bruises, epistaxis, or any other sign of bleeding.

**Table 1:** Historical platelet trending

Platelet Count	
Date	Value (k/μL)
Four days before the vaccine	261
Four days after the vaccine	250
14 days after the vaccine (visit to other hospitals)	8
Admission Day 1 (21 days after the vaccine)	11
Admission Day 2	63
Before discharge	122
Clinic follow up	387

She received a blood transfusion with no change in her platelet count. She was evaluated for thrombocytopenia and was found to have HIV, HCV, and Coombs test negative. Antinuclear antibodyANA was reactive (1:40). Computed tomography (CT) of the abdomen and pelvis did not reveal cirrhosis or spleen abnormalities. The peripheral smear did not show platelet aggregation or fragmentation of RBCs.

The patient was immediately started on IV gammaglobulin (IVIg) 1g/kg once a day (qd) for two days and dexamethasone 20 mg IV every 12 hrs (q12h). The next day, the platelet count increased to 63 k/ul, and after the second dose of IVIg, the platelet count improved to 122 k/ul and trended up (Table 1).

## Discussion

Immune thrombocytopenic purpura and immune thrombocytopenia could represent life-threatening conditions and require immediate diagnosis and subsequent treatment [5]. This condition could be related to an autoimmune reaction due to molecular mimicry due to specific autoimmune conditions, acute infections, or the immunization process [6].

The world faced the greatest scientific challenge in recent years due to the COVID-19 virus. ITP was brought back into the public eye over the last years with the advent of COVID-19 vaccines to fight the global pandemic caused by severe acute respiratory syndrome coronavirus 2 (SARS-CoV-2). From the literature, there are 17 cases to date that link thrombocytopenia to COVID-19 vaccines (AstraZeneca, Moderna, and Pfizer), which has been noted after two to three weeks of the vaccine. Ours is the 18th case of thrombocytopenia due to the COVID-19 vaccine and only the 10th case due to the Pfizer vaccine [7,8].

Multiple well-known laboratories started working on the vaccine and achieved their goal, but the mechanism of the vaccines was different, in our country, three vaccines were approved: two of them used the mRNA (Pfizer and Moderna) and one was a viral vector (Johnson & Johnson) [9-10]. Their side effects were directly related to the allergy to one or more of their component. Another vaccine that is not approved by the FDA but has been widely used in other countries is the ChAdOx1 nCoV-19v (AstraZeneca), which is a viral vectored coronavirus vaccine, that expresses the spike protein of SARS-CoV-2 [11].

The Centers for Disease Control established that the immunization process after the COVID-19 vaccine is completed after two weeks of the completion of the single-injection vaccine (Johnson & Johnson) or two weeks after the second dose of the two injections vaccine (Pfizer and Moderna) [12].

The ChAdOx1 nCoV-19 has been related to other serious side effects, including embolic events after the immunization process, but there are less than 100 patients after 17 million vaccinated patients [13]. On the other hand, the Johnson & Johnson vaccine has been related to a similar side effect in a similar number of patients, this is why the FDA stopped the administration of this vaccine but later re-started it, advising that “Women younger than 50 years old should be aware of the rare risk of blood clots with low platelets after vaccination” [14].

During our literature review, we found only a few articles reporting thrombosis and thrombocytopenia after administration of the ChAdOx1 nCoV19 vaccine (AstraZeneca) related to the AstraZeneca vaccine [15,16]. On the other hand, we have 20 cases of thrombocytopenia post-Moderna (11 cases) or Pfizer (nine cases) vaccination, 17 of them did not have a history of thrombocytopenia while 14 had bleeding symptoms before their hospitalization [17]. The mechanism of this phenomenon is not completely understood but one of the hypotheses is the presence of autoimmune Heparin-Induced Thrombocytopenia (aHIT), indicating the presence of anti-platelet factor 4 (PF4)-polyanion IgG antibodies activating the platelets in the absence of heparin [18]. Other possibilities include the possible pre-formed antibodies (Ab) against poly‐ethylene‐glycol or other lipidic components or antibody-mediated pro-coagulant platelets [19].

The exact mechanism and cause of thrombocytopenia is not fully understood. These patients have been treated with steroids and IVIg. In some cases, rituximab has been used to decrease the immune mechanism against platelets, to avoid complications and improve patient outcomes [20].

## Conclusions

Immune thrombocytopenic purpura induced by mRNA COVID-19 vaccine is uncommon. Its early detection could represent a challenge for our clinicians. A prompt and complete individualized assessment would be a determinant to guide an early and directed therapy with gammaglobulin and steroids to improve the outcome of our patients.
